# Deep learning-based extraction of Kenya’s historical road network from topographic maps

**DOI:** 10.1038/s41597-025-05442-6

**Published:** 2025-07-05

**Authors:** Tanja Kramm, Nicodemus Nyamari, Vincent Moseti, Annika Klee, Leon Vehlken, David M. Anderson, Christina Bogner, Georg Bareth

**Affiliations:** 1 GIS and Remote Sensing Group, Institute of Geography, University of Cologne, 50674 Cologne, Germany; 2 Ecosystem Research Group, Institute of Geography, Faculty of Mathematics and Natural Sciences, University of Cologne, 50674 Cologne, Germany; 3 Center for Development Research (ZEF), University of Bonn, 53113 Bonn, Germany; 4 Department of History, University of Warwick, Faculty of Arts Building, Coventry, CV4 7EQ United Kingdom; 5 Global South Studies Center, University of Cologne, 50931, Cologne, Germany

**Keywords:** Environmental impact, Environmental impact, Environmental economics, Ecological modelling

## Abstract

Kenya’s road network significantly influences environmental and socio-economic dynamics. High-quality road data is essential for analyzing its impact on various factors, including land-use, biodiversity, migration, livelihoods, and economy. Like many countries, Kenya faces challenges in the availability of accurate and detailed digital historical road datasets. To address this, we used deep learning techniques to extract Kenya’s road network from 533 historical topographic maps (1:50,000 and 1:100,000 scale) covering the 1950s–1980s. This involved digitizing, georeferencing, and classifying of 20 different road symbols on all maps, then converting and merging them into a seamless dataset. The statistical evaluation was conducted against manually created roads from seven representative map sheets by calculating precision, recall, and F1 score. Our study provides a detailed historical road dataset for Kenya containing over 56,000 km of historical roads. The statistical validation showed an average F1 score of 0.84, indicating a high classification performance. The methodology offers an applicable approach for national-level historic road network mapping, also transferable to other regions, map types or features.

## Background & Summary

Roads play an important role in Sub-Saharan African (SSA) countries for the transportation of people and goods^1,2^. They connect cities and facilitate movements over larger distances^3,4^. Therefore, they are often considered as a main driver of development and modernity that enables the access to markets and job opportunities, as well as basic social amenities, such as healthcare services and education^5–7^. However, besides these mentioned positive aspects, the development of roads often also brings a multitude of negative consequences for ecosystems, livelihoods, and biodiversity. Several studies have found that roads increase deforestation^8^, reduce species diversity^9^, contaminate soils^10^, increase human–wildlife conflicts^11–13^, raise socio-economic inequalities^14^, and human health risks^15,16^.

The multifaceted impacts of roads are complex, and their research involves a wide range of different disciplines and times. Observing road development over longer time requires highly accurate and complete road network data from different time periods. The use of incomplete or inaccurate datasets can have a negative impact on the quality of analyses and lead to misleading interpretations^17,18^. Most countries of the world lack appropriate road network data across different time periods, as their mapping authorities are facing several financial, legal and technical barriers to create road network datasets over larger areas and keep them up-to-date^19–21^. This applies in particular to the era before Open Street Map and other publicly created datasets became available. To overcome this issue, many researchers have achieved satisfactory results in extracting road information from modern high-resolution satellite imagery^22–26^. Others have not extracted historical road networks, but used modelling approaches to backdate the contemporary road network and create time series reflecting the urban sprawl of road networks over time^27–30^. These approaches measured urban sprawl by using the mean nodal degree of intersections as a measure of connectivity of the road network. However, these modelling approaches have the disadvantage compared to the actual extraction of the historical road network that they can only model growth, but cannot identify potential shrinkage of the road network. Extracting historical road information further back in time is still challenging given the lack of appropriate available imagery or other geospatial data. Historical geographic data is often available on printed maps only that are neither scanned nor georeferenced^31^. Nevertheless, these maps contain a huge untapped potential of existing historical geodata, which could be made digitally available using modern methods of geoinformatics and remote sensing. These data would offer the possibility for researchers to gain insights into the historical evolution of landscape and infrastructure and enable long-term studies over larger areas^32,33^.

Extracting complete and accurate historical road datasets is still a challenging task. A manual extraction of geographic information from historical maps is a very time-consuming approach that is not feasible for a larger area^34^. Therefore, the development of automatic approaches to extract information from digitised historical topographic maps is necessary to realise the potential that lies in the available information contained in these maps^35,36^. Several studies have made major advances, developing methods to automate the extraction of information from these datasets. In particular, the detection and extraction with deep learning techniques have been proven as a promising approach. For example, Can *et al*.^34^ extracted seven different road types from a set of Military maps conducted between 1884 and 1918 with a deep learning CNN-based model. A region-based deep convolutional neural network (RCNN) method was used by Saeedimoghaddam *et al*.^37^ to detect road intersection points from the USGS historical topographic map collection (mostly 1:100,000). A weakly supervised CNN framework was trained by Uhl *et al*.^38^ to classify roads and buildings from the USGS historical maps (1:62,500). Ekim *et al*.^39^ classified five different road types from the Deutsche Heereskarte (1:200,000) for Turkey with a proposed deep-neural network U-Net++ architecture and Mäyrä *et al*.^32^ used a U-Net classifier to extract roads and other features from historical scanned maps for a small area in Finland.

The history of roads in Kenya is closely linked to the country’s development and its colonial past. Before colonialism, mobility was defined by caravan tracks used by ivory and slave traders. The first formal road to be constructed under colonialism was Sclater’s Road, a rough waggon track from Mombasa to the shores of Lake Victoria at Kisumu^40^. However, road development was slow, with motorized journeys beginning only in 1926. Early road development focused on urban centers, with bitumen roads appearing in major towns by the 1930s, while rural areas had dirt tracks maintained through forced labor^41^. Overall, road development was highly biased towards connecting urban centres and small towns, mainly created during the colonial period to connect administrative headquarters and cutting travel times^42^. These roads were symbols of colonial oppression and often became invariably overgrown and impassable if not in regular use^43^. Only with the growth of commercial opportunities did rural communities begin to view the road network in a more positive way^41^. This was influenced by local economic development, but could also be linked to issues of ecology, such as the presence of wildlife^44^. In recent decades, initiatives like “Kenya Vision 2030” increased investments in infrastructure, which has led to a considerable expansion of the road infrastructure^45^. However, challenges such as inadequate maintenance, insufficient funding, and traffic congestion remain.

Like in many other countries, digitized road information on the past is hardly available in Kenya. The country holds a rich collection of historical topographic maps that cover the nationwide road network from the 1940s-1980s. The British Overseas Collection produced numerous maps from this era, which are to date available at several archives in Kenya and the UK. These maps contain a wide variety of geographical information about Kenya’s landscape and infrastructure from that time. Besides an effort by Princeton University to create a dataset of Africa’s road network from the 1960s, no nationwide digital historic road data from the mid-20th century is available^46^. However, their dataset only contains major roads on a very broad scale extracted from the Michelin Africa maps atlas at a scale of 1:4,000,000, which is unsuitable for any kind of large-scale analysis.

Historical maps contain a rich collection of geographic information that are barely usable for modern research approaches in their analogue form. The goal of our study is to make the historical information available in several archives usable for modern digital analysis methods and tools by extracting the road network for the whole of Kenya from historical topographic maps. We derived detailed road network information from over 500 available historical maps at scales of 1:50,000 and 1:100,000 that differ greatly in terms of their appearance, symbolism, and scanning quality. We use deep learning methods to detect the roads printed in the maps and convert the analogue information into digital GIS formats to create for the first time a highly detailed digital dataset of Kenya’s road network that reflects its mid-20th-century development stage. The dataset is created within the framework of the research project CRC/TRR228 “Future Rural Africa: Future-making and social-ecological transformation” to support further research on synergies and trade-offs of road development on rural communities, biodiversity and ecosystem services in Kenya. It is provided openly available in the CRC/TRR228 project database, the TRR228DB. The dataset enables opportunities for long-term research that requires a historical perspective, for example the analysis of Kenya’s infrastructure evolution and its potential consequences on various environmental and socio-economic developments. To evaluate the overall quality of the extracted dataset, the statistical measures of overall accuracy, precision, recall, F1 score and Intersection over Union (IoU) are calculated by comparing the extracted historical road network against seven representative map sheets with a manually digitized road network. To evaluate the spatial accuracy of the extracted road network, we determined the positional deviations of intersections present in the extracted road network and in the current road dataset of the Kenyan Roads Board (KRB), and calculated the root mean square error (RMSE) and the Mean Absolute Deviation (MAD) for each of the two map scales.

## Methods

### Collection of Historical Topographic Maps

The topographic maps used in this study were obtained from various sources in Kenya and the UK. All maps were created and published between the 1950s and early 1980s^47,48^. The majority of maps were collected in Kenya from the Survey of Kenya as the official custodian and authority for Kenyan topographic maps. Additionally, maps were obtained from different local county governments’ survey and urban planning departments. A total of 449 1:50,000 scale maps and 71 1:100,000 scale maps could be collected from Kenyan sources. However, 166 of the 1:50,000 maps and 94 of the 1:100,000 were only available as data frame clips with no further information on the year of publication and source. In addition, some maps were technical drawings rather than fully processed topographic maps and had a notably different standard than the other maps. Nevertheless, these maps also showed road networks, making them suitable for the study’s purposes.

**Fig. 1:**
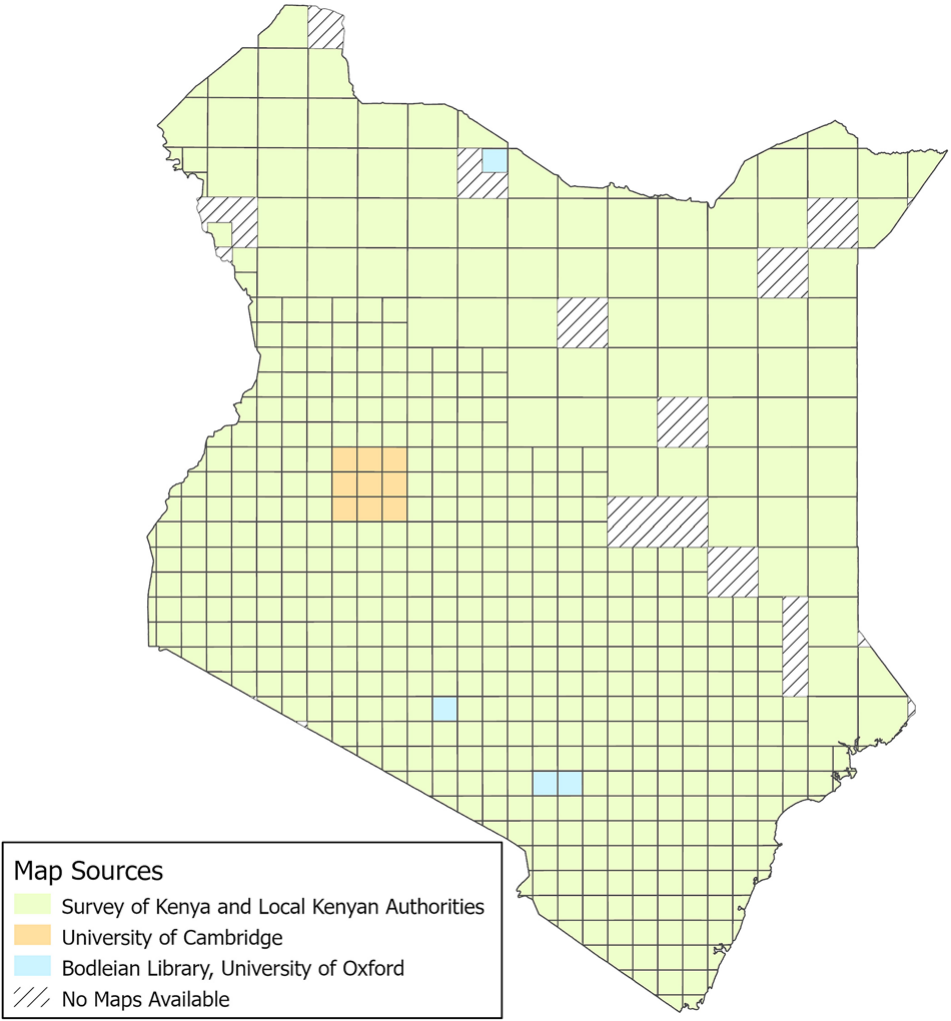
Source and location of the topographic maps used in this study covering the time period between the 1950s and 1980s. Each square represents a single map sheet. Large squares correspond to maps with a scale of 1:100,000, smaller squares to maps with a scale of 1:50,000.

**Fig. 2:**
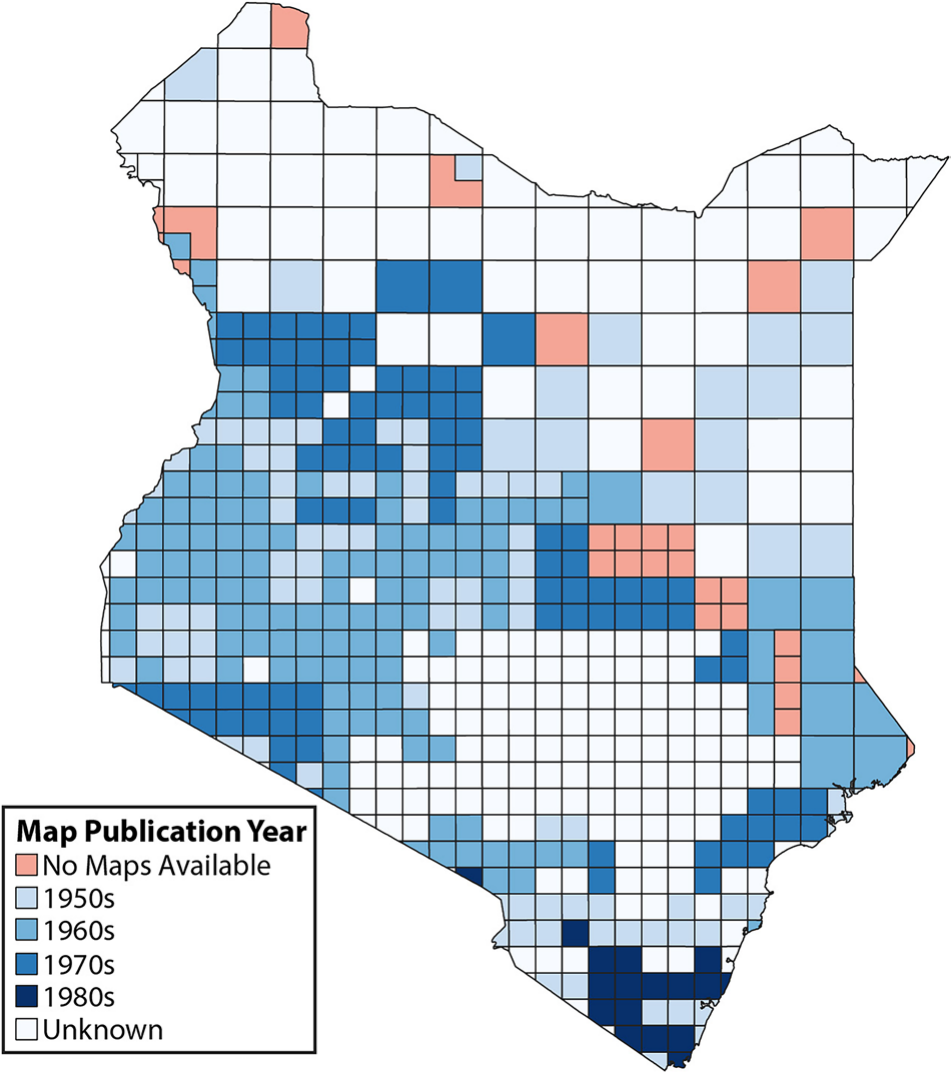
Publication years of the topographic maps used in this study. Each square represents a single map sheet. Large squares correspond to maps with a scale of 1:100,000, smaller squares to maps with a scale of 1:50,000.

As it was impossible to get all the necessary sheets to achieve complete coverage of the country from the Survey of Kenya, several additional maps were purchased from archives in Great Britain. These British archives contain historical maps originally created and published by the Directorate of Overseas Surveys (DOS) and the War Office, General Staff, Geographical Section. A total of nine maps were purchased from the Cambridge University Library and four maps were used from the Bodleian Libraries of the University of Oxford. Figure 1 provides an overview of all the acquired map sheets. Unfortunately, for an area of 31,400 km^2^, no suitable maps could be obtained for this study. The publication year of each map sheet is depicted in Fig. 2. All maps used in this study are not accessible online and are subject to the licences of the aforementioned British archives and the Survey of Kenya.

### Provenance of the Historical Maps

The Survey Department established in Kenya’s colonial capital, Nairobi, in 1903, first began work to register land claims made by prospective white settlers, and to issue deeds to commercial enterprises. Later renamed as the Survey of Kenya, by the 1950s the department had a technical Training Wing and a large African Lands Division devoted to mapping consolidated plots of fragmented African rural land^49^. The government department still functions today at Ruaraka, and is responsible for geodetic, topographic, photogrammetric and cadastral surveying, and the publication of all official topographic and cadastral maps of Kenya. However, because of government controls, maps produced by the Survey of Kenya were difficult to obtain in Kenya until after 2000, and the public relied predominantly upon the commercially-produced Michelin Motoring Map Series 746 (and its predecessor Series 155)^50^.

The Survey of Kenya has historically seen its role as servicing the government, rather than the public. From the 1920s onwards, the Survey of Kenya enjoyed a good reputation for ground surveying, but the accuracy of the maps produced came to be reflected in their basis in extensive aerial survey, which began to be utilised in the late 1930s and became the norm after the Second World War^51^. The war also brought about far greater coordination of map-making across the British Empire, and led directly to the creation by the Colonial Office of the Directorate of Overseas Surveys (DOS). From 1946, this body was responsible for mapping in all British dependencies globally. The DOS contracted wide-ranging aerial surveys of each country, to provide a basis for cartography. Initially, air surveys in Kenya were undertaken by local aviation companies, such as Spartan Air Services (Eastern) Ltd, but by the end of the 1960s the job was being contracted to larger professional consultancy firms, such as Hunting Technical Services^52^.

The DOS operated independently, under the auspices of the Colonial (and then Foreign & Commonwealth) Office until 1984, when it merged with the British Ordnance Survey (OS). Throughout its existence, the DOS worked closely with the Survey of Kenya, producing annual reports that detail its activities in the former colony^49^ – these can be consulted in series OS 46 at the British National Archive, Kew^53^. The involvement of DOS in conducting aerial surveys continued after Kenya’s political independence in 1963. After 1984, DOS became known as the Overseas Surveys Directorate, operating within the UK’s Ordnance Survey department. It continued to function without much change until 1991, when the last significant aid-funded mapping projects in the former British colonies came to an end. At this point, the colonial ties were finally cut, and the name was changed yet again, to Ordnance Survey International (OSI), which now functioned as a commercial consultancy^52^.

From 1991, there was uncertainty about how, and where, the archive of the DOS and its successor should be housed. Over the next decade, many elements of the archive became scattered in libraries and other depositories around the UK – for example, at the Bodleian Library in Oxford. Only when Ordnance Survey International finally ceased to function in 2001, did responsibility for its records, and for the maintenance of the historical maps, finally pass to the UK National Archive. The collection of maps had been stored as a working collection with the Ordnance Survey in Southampton, but under Ordnance Survey International this was partially moved to the British Empire and Commonwealth Museum in Bristol. When this museum became defunct, the cartography collection eventually (2012) found a new home in the National Collection of Aerial Photographs, under the management of Historic Environment Scotland^52,53^.

The splintering of the UK overseas survey functions after 1991 coincided with a very difficult period of under-funding and administrative neglect for the Survey of Kenya, when the archiving of their own collection of maps fell into disarray – a situation that has only improved again in very recent years. This fragmented history of institutional change helps to explain why Kenya’s historical maps are so scattered among several incomplete collections. In a final complexity, the correspondence files relating to the creation of each series of maps that should accompany them, are stored separately at The National Archive, Kew, as part of the Overseas Development (OD) series^53^.

### Scanning & Georeferencing

All available analogue maps were scanned into high-resolution raster data for further processing. All digitized maps were then imported into ArcGIS Pro 3.2 and manually georeferenced to the Universal Transverse Mercator (UTM) projection using Arc 1960 as the geodetic datum. As Kenya lies both to the south and the north of the equator and is covered by zones 36 and 37, the maps were projected to the coordinate systems Arc_1960_UTM_Zone_36N, Arc_1960_UTM_Zone_37N, Arc_1960_UTM_Zone_36S, Arc_1960_UTM_Zone_37S depending on their location. As reference grid for georeferencing, a Topographical Index Grid shapefile, produced by the Survey of Kenya for both the 1:50,000 and 1:100,000 scales, has been used. The Index Grid contains the sheet number of the respective topographic map and consists of regular quadrangles fitting to the coordinates of the map frame boundaries. We always used one map sheet per grid tile to avoid any overlap of map sheets. For maps that provided further coordinate information and contained a coordinate grid within the map, further reference points were set across the map to increase positional accuracy. Depending on the number of control points set, a 1st-, 2nd-, or 3rd-order polynomial transformation was used by opting for the one providing the smallest calculated residuals.

### Training & Classification

**Fig. 3:**
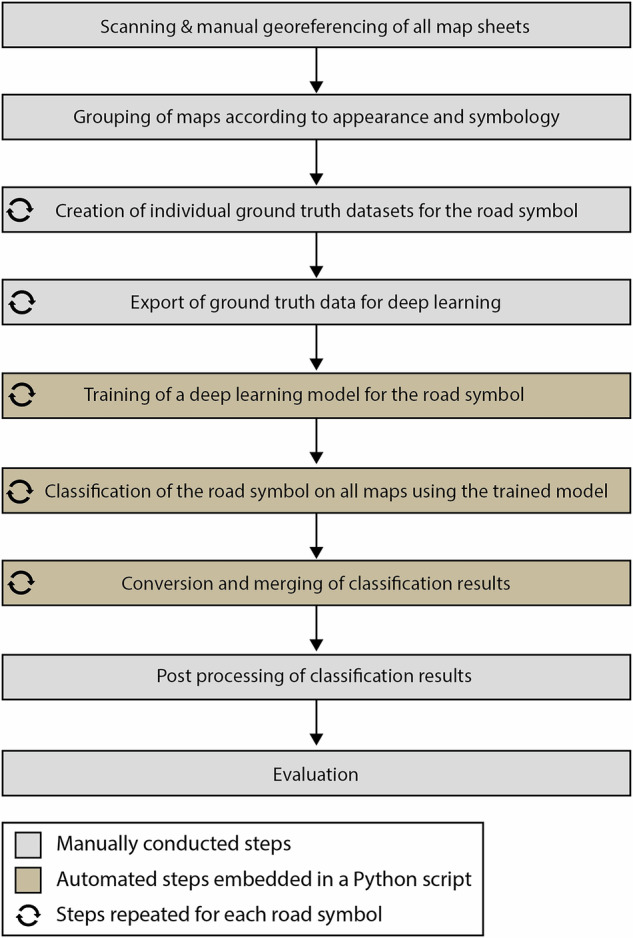
Schematic workflow conducted in this study to extract historical roads from topographical maps.

**Fig. 4:**
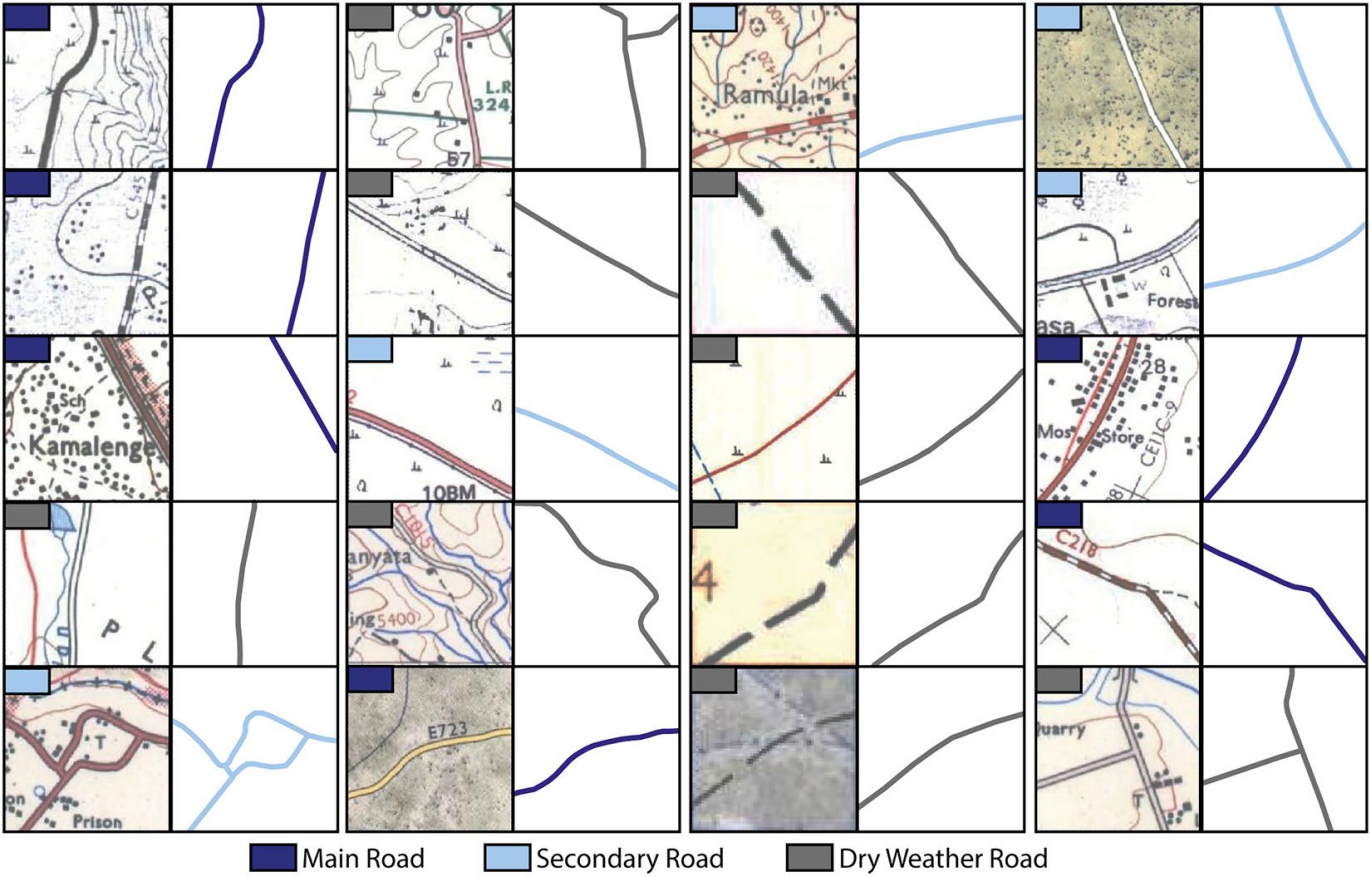
Different road symbols that appeared in the used topographic maps and had to be extracted separately. Six road symbols belong to the category “Main Road with Bound Surface” in the historical maps, five symbols to the “Secondary Road with Loose Surface” category and nine symbols are representing the category “Dry Weather Road/Motorable Track”.

Several methodological steps were necessary to perform the road extraction. The overall workflow is presented in Fig. 3. In the initial preparation step, the maps were sorted into different groups according to their map style, as they greatly differed in terms of appearance and symbology. Overall, the maps contained three different types of roads (Main Road, Secondary Road, Dry Weather Road) according to their symbology. However, since the maps were from various sources, editions and years, a road class was represented by different symbols on different maps. For example, main roads were shown in dark red lines on one map and in black lines on another map of different edition or source. Subsequently, each individual road symbol from each map group has to be trained and classified separately. In total, 20 individual classifications had to be performed to classify all different types of road symbology (Fig. 4).

Before model training, a ground truth dataset was manually created for each road symbol in ArcGIS Pro over the roads shown in the georeferenced maps. To match the width of the roads on the map, a 20 m buffer was created around all digitized road lines. For the deep learning model training, it is crucial that the buffer width fully encompasses the entire road symbol, necessitating a buffer width that is slightly wider than the symbol itself. In our case, a buffer width of 20 m turned out to be appropriate for all used road symbols. It was important that the set of roads covered be as representative as possible, including all the different types of road representation on the maps, covering horizontal and vertical roads, very curvy and straight roads, and different types of road intersections. Following the initial preparation, the polygons were extracted into image chips along with corresponding labels using the ArcGIS Pro 3.2 tool “Export training data for Deep Learning”. This step ensured that the data adhered to the format required for subsequent deep learning model training. The parameters of the tool were set to automatically produce labels and image tiles of 512 × 512 pixel size with an overlap of 128 × 128 pixels. Setting an overlap of images increases the number of extracted image tiles, but overall reduces information loss during the deep learning training process. However, higher overlaps require more computational power and extend the overall computation time. The chosen value here represents a balance between these competing factors and proved to be the best compromise in our case between achieving high model accuracies and maintaining reasonable computation times. The output was exported in TIFF file format using the Metadata Format “Classified Tiles”. Depending on the available length of each road type, a total of 900 to 8400 image tiles were extracted as training. The export of training data for maps of both scales could be treated equally using the same workflow and values.

In the next step, the exported training data was used to train the deep learning model and perform the classification for all individual road types. This was done in a Python script to automate the process of model training and the classification of the trained road type across a large number of maps where the specific road type occurred^54^. The ArcGIS Pro deep learning-based “Multi-Task Road Extractor” tool was utilized for model training using a ResNet34 deep learning architecture, which is a convolutional neural network (CNN). The tool was set with “hourglass” architecture, using a U-Net model for image segmentation. An optimal learning rate was calculated before model training. A maximum of 50 learning epochs was set, with optional early stopping when model accuracy plateaued. To test the accuracy of each model, a split of the exported training data in 90% training and 10% test data was done within each model training by the “Multi-Task Road Extractor” tool and the mean Intersection over Union (mIoU) was calculated for each trained model. All trained models in this study had a mIoU of 0.992 or higher indicating a high performance of the trained deep learning models. The successfully trained model was then used to classify the trained road type on all maps using the ArcGIS Pro “Classify Pixels Using Deep Learning” tool, resulting in binary classifications of all individual roads across all available maps.

### Postprocessing

Subsequently, the extracted roads were transformed from a raster dataset to editable polygon vector data allowing further dataset processing. To address small misclassifications outside of roads and fill small holes within the classified roads, the polygons were simplified with the tool “Simplify Polygon” and then converted into a line shapefile using the “Polygon to Centerline” tool. The objective was to create a seamless dataset from all classified pieces of roads by merging all classified road segments and assigning the road class to each individual road as an attribute. The resulting set of roads encompassed three distinct road classes based on the road symbology in the original maps, main roads with bound surface, secondary roads with loose surface and dry weather roads that are suitable for motorable off-road vehicles under favourable weather conditions.

To improve the overall classification accuracy, several post-processing steps were conducted. This involved the manual refinement of any remaining misclassifications and adding any road segments that were initially missed by the classifier. Furthermore, topological checks have been done to identify over- or undershooting lines to create a seamless dataset. To improve the topological integrity of the dataset, the ArcGIS Pro tools “Trim Line” and “Extend Line” have been used to automatically eliminate the majority of topological issues and to create connected intersections. Additionally, the dataset has been manually revised to eliminate remaining issues.

### Validation

To assess the accuracy of the classified roads, several evaluation metrics were computed. First, the overall accuracy was calculated from confusion matrices of the portion of correctly and falsely classified road and non-road areas. Consequently, the common evaluation metrics precision (often also referred to as correctness) and recall (often also referred to as completeness) scores were calculated. Precision is a widely used metric in machine learning evaluation that quantifies the proportion of true positive predictions out of the total number of positive predictions made by the model. Essentially, it measures the model’s ability to avoid false-positive predictions. Recall is the ratio of correctly predicted positive instances to the total number of actual positive instances. In simpler terms, recall measures how effectively the model can detect positive instances, even if it occasionally misclassified some negative instances as positive. Both metrics can be calculated as follows:

1 $$\,{\rm{Precision}}=\frac{{\rm{True\; Positive}}}{{\rm{True\; Positive}}+{\rm{False\; Positive}}}$$

2 $$\,{\rm{Recall}}=\frac{{\rm{True\; Positive}}}{{\rm{True\; Positive}}+{\rm{False\; Negative}}}$$

By combining the scores of precision and recall, the F1 score is a widely used metric for assessing the performance of classification models. It is calculated by computing the harmonic mean of precision and recall using the following formula:

3 $$\,{\rm{F1}}=2\times \frac{{\rm{Precision}}\times {\rm{Recall}}}{{\rm{Precision}}+{\rm{Recall}}}$$

Additionally, we have calculated the intersection over Union score that is beneficial to analyse the overlapping level of the classified road segments. It divides the overlapping area between the classified and ground truth dataset by the total area covered by both datasets and is calculated using the following formula:

4 $$\,{\rm{IoU}}=\frac{{\rm{Area\; of\; Intersection}}}{{\rm{Area\; of\; Union}}}$$

**Fig. 5:**
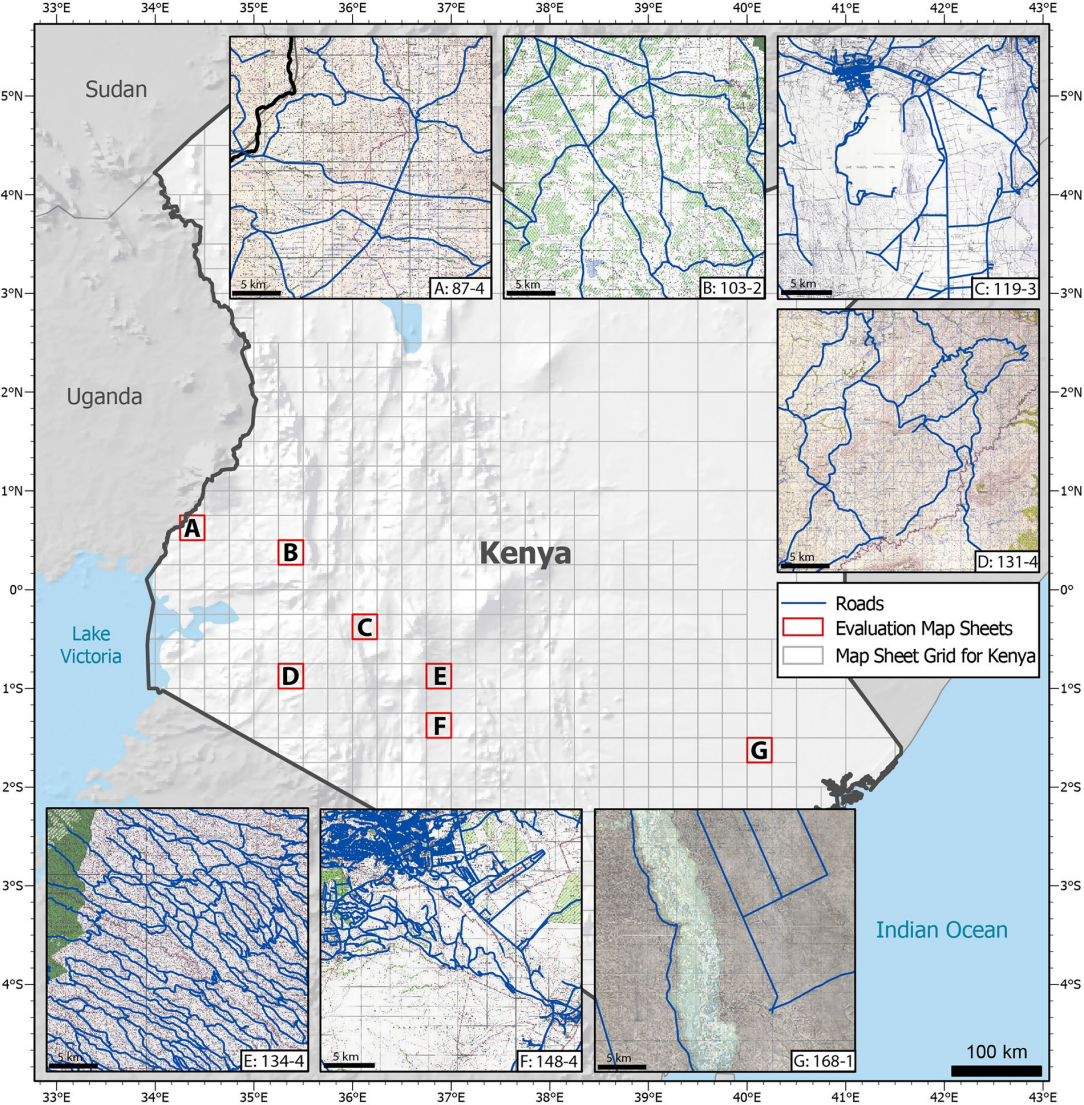
Overview and location of the seven maps (labelled with their sheet number) and the created ground truth data used for accuracy analysis.

All described metrics yield values ranging between 0 and 1. A value of 1 indicates perfect accuracy, while a value of 0 signifies an overall poor classification result. These metrics were calculated for the classified roads without further preprocessing on seven representative map sheets that are significantly varying in terms of map appearance and the depicted landscape. As a ground truth dataset, the road network of these map sheets has been drawn manually to perfectly match the displayed roads on the map. This ground truth road network was then compared with the uncorrected classification result, which was obtained from the classification process without any subsequent manual improvements. Since both datasets were created using the same maps, these accuracy values evaluate the classification accuracy only without considering the positional accuracy of the extracted roads. To consider the average width of roads shown on the maps, both datasets were compared using a calculated buffer of 20 m. The selected map sheets and their respective locations are depicted in Fig. 5.

Additionally, we have assessed the positional accuracy of the extracted road dataset to account positional inaccuracies introduced by the maps themselves or during the georeferencing process. The assessment has been conducted by comparing spatial deviations of road intersections that already existed in the historical dataset and appear to be largely unchanged in a recent road dataset of Kenya. As reference data, we used a current road dataset provided by the Kenyan Roads Board^55^. This dataset is not openly accessible, but was provided to us by the KRB on request. To assess the spatial accuracy, 100 reference points were identified on the maps with a scale of 1:50,000 and 44 points were used on the 1:100,000 scale maps. Based on the deviations that we determined at these points between the intersections in both datasets, an RMSE and an MAD was calculated for each map scale.

## Data Records

**Fig. 6:**
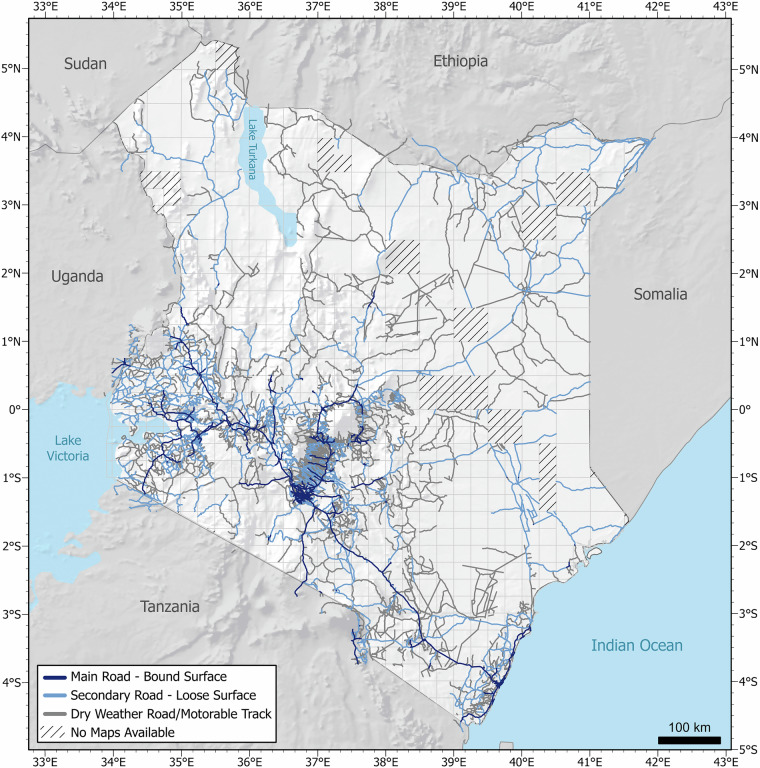
Extracted historical road network of Kenya from topographic maps covering the time period ranging from 1950s to 1980s.

The historical road network dataset of Kenya consists of a polyline shapefile in the ESRI Shapefile format. It is publicly available in the data repository of the CRC/TRR228^56^. The resulting road network of the entire country is shown in Fig. 6. In total, 56,238 km of roads were extracted. Each road segment contains the attributes listed in Table 1.

**Table 1:** Attributes assigned to the historical road network dataset of Kenya.

Attribute	Data Type	Description
**FID**	Object ID	Unique ID for each road segment.
**Road Type**	Integer	The type of road, represented as an integer, derived from its categorization on the corresponding topographic map.
**RoadName**	Text	Full name of the road type, derived from its categorization on the corresponding topographic map.
**MapYear**	Integer	Publication year of the topographic map, where the road was extracted from. If the value is NULL, the published year of the map is unknown, meaning the map was available as a clip of the data frame only without marginal information.
**SHEET_NO**	Text	Sheet number of the topographical map as assigned by the Survey of Kenya.
**Length**	Double	Length of the road segment in meters.

The classified roads were categorised into three distinct classes based on the road symbology in the original maps. In rare cases where maps had only two classes, the road segments were assigned to one of the three classes using expert knowledge. A total of 4,845 km was identified as “Main Road with Bound Surface”, 19,540 km of road segments were identified as “Secondary Road with Loose Surface”. 31,853 km of road segments were categorised into the third category “Dry Weather Road/Motorable Track”. Consequently, the majority of Kenyan historic roads consisted of loose surfaces and only approximately 8.6% of the classified roads fall into the primary road category with a bound surface. 34.8% of Kenya’s historical road network fall into the secondary road category consisting of a loose surface, and 56.6% of roads fall into the third category of roads that were only passable during dry weather and with suitable off-road vehicles. The dataset is projected to the “Arc_1960_UTM_Zone_37N” (EPSG:21097) coordinate system.

## Technical Validation

**Fig. 7:**
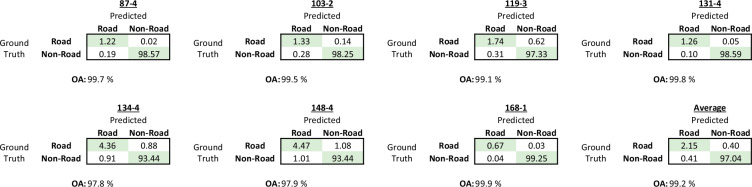
Confusion matrix and calculated overall accuracies (OA) for the seven ground truth map sheets. The proportion of correctly and incorrectly classified road areas (in percent) was determined by comparing them to the manually generated ground truth data.

The quantitative accuracy assessment of the achieved results is presented in Fig. 7 and Table 2. The extracted roads from seven map sheets (in the following labelled by their index numbers) were statistically evaluated by calculating the overall classification accuracy, as well as precision, recall and F1 score of each individual map sheet. The results of Fig. 7 show for each tested map sheet the confusion matrix of the percentage of road and non-road areas classified correctly and incorrectly by comparing them to the manually created ground truth data. The achieved overall accuracies show an average accuracy of 99.2% over all tested map sheets ranging between 97.8% for map sheet 134-4 and 99.9% for map sheet 168-1. The calculated F1 scores in Table 2 indicate an overall high classification accuracy, with values ranging from 0.79 for map sheet 119-3 to 0.95 for map sheet 168-1. The achieved average F1 score across all tested map sheets was 0.84. Likewise, the lowest IoU was calculated for map sheet 119-3 and the highest IoU for map sheet 168-1. The average IoU that has been calculated across all seven tested map sheets was 0.73. The differences in accuracy between the different map types were only minor. Overall, coloured maps tended to achieve slightly higher classification results compared to black and white maps. Likewise, more complex road layouts in mountainous areas (map 134-4) or cities (map 148-4) led to a decrease in classification accuracy. The roads on map 119-3, which had a slightly poorer scan quality and a blueish tint from the scanning process, generally achieved the lowest accuracies. Nevertheless, the observed accuracy of extracted roads remained high across all tested map sheets.

**Table 2:** Results of the calculated accuracy measures precision, recall and F1 score obtained for the area of seven map sheets to evaluate the accuracy of the classified roads. The values were calculated using a buffer of 20 m to mitigate small positional misalignments of both datasets.

Sheet number	87-4	103-2	119-3	131-4	134-4	148-4	168-1	Average
**Precision**	0.86	0.83	0.85	0.93	0.83	0.82	0.94	0.84
**Recall**	0.99	0.90	0.74	0.96	0.83	0.81	0.95	0.84
**F1**	0.92	0.86	0.79	0.94	0.83	0.81	0.95	0.84
**IoU**	0.85	0.76	0.65	0.89	0.71	0.68	0.90	0.73

The analysis of the positional accuracy of the extracted road network based on the spatial deviations of intersections between the historical and a current road dataset revealed significantly different results between both map scales. For the 100 intersections on the 1:50,000 maps, an RMSE of 17.8 m has been calculated. The calculated MAD of this map scale was 13.0 m. In contrast, the deviation on the 1:100,000 maps appeared to be significantly higher with an RMSE of 245.1 m and a MAD of 202.8 m calculated over 44 intersections, indicating an overall lower spatial accuracy of the extracted roads in the northern part of the country, which is mainly covered by maps of this scale.

### Harmonization and Data Quality

Most roads were classified accurately and completely across various parts of the maps. About 5.4% of the country could not be classified, as we were unable to obtain maps for these areas. Consequently, some road segments passing through these unmapped regions may appear as broken links. However, given that most of the missing map tiles are in rural areas, the overall proportion of missing road kilometres is likely to be small.

The spatial accuracy of the extracted road dataset highly depends on several factors. Significant spatial deviations can occur due to inaccuracies in the georeferencing process. Furthermore, the positional accuracy of the extracted roads is highly dependent on the overall quality of the map itself, as cartographic generalisation and insufficient data of which the map was created can lead to errors or positional inaccuracies that are not necessarily classification errors, but rather already occur in the original maps. Furthermore, distortions that occur during the scanning process or due to bad condition of a poorly stored map can lead to severe positional inaccuracies of the extracted roads. The overall achieved positional accuracy for the roads extracted from the 1:50,000 map sheets can be considered as generally high as they lie within the map accuracy standards of 25.4 m proposed by the USGS for this map scale^57^. However, the analysis of the 1:100,000 maps reveals overall much larger deviations. The achieved positional accuracies for this map scale are significantly lower than the accuracy standards of 50.8 m or less for maps of 1:100,000 scale^57^. Since we have used the same georeferencing workflow and tools for all maps of both scales, we assume that the observed differences in RMSE values stem primarily from inaccuracies in the maps themselves. It can be assumed that these inaccuracies are likely caused by a higher degree of generalization in these maps and also probably due to less accurate data available in the northern part of Kenya to create the maps. However, it cannot be exactly determined within this study to which degree these inaccuracies are already present in the original maps or induced by inaccuracies during the georeferencing process.

During this study, several challenges emerged, impacting the harmonization and quality of the results, leading to inconsistencies in the classification of road types. Several issues arose from the great heterogeneity of the maps obtained from diverse sources to achieve an almost complete country coverage. Therefore, the maps strongly varied in terms of their appearance, scanning quality, and road symbology. Numerous different map editions resulted in various different symbols representing different road types. Contextual inconsistencies in the road classification arose from map sheets covering different time periods and showing different states of the road between neighbouring sheets. This resulted in roads that sometimes changed their category or disappeared abruptly when transitioning to another map sheet. Reasons could be that the road did not exist or was in a different condition in the following map sheet from an earlier time period. Furthermore, different editions of neighbouring map sheets could lead to different road categories due to variations in legend divisions. In summary, these issues resulted in multiple inconsistencies within the road dataset and, in rare instances, caused the creation of orphaned roads that have no connection to other roads.

Upon classifying all map tiles, the extracted roads at the map boundaries often do not align perfectly, depending on the georeferencing accuracy across the maps. While small gaps can be automatically connected, larger gaps required manual adjustments and connections. The same challenge arose at road junctions where different road types intersect. To achieve a seamless road network, these small gaps had to be manually removed afterwards. Despite the availability of some tools for automatic gap refinement, they do not always resolve all topological issues comprehensively. Consequently, the manual refinement of the extracted road network has been proved time-consuming, especially given the large area covered by this study. Approximately 25% of the roads included parts that were either completely missing or insufficiently classified, and required manual adjustment, or were incorrectly classified and needed removal. However, this proportion can vary significantly on a map by map basis depending on the complexity of the road network or the characteristics of the landscape shown on the map. Furthermore, it can be assumed that not all issues have been resolved by the manual correction and a certain number of inconsistencies still exist in the dataset. We went through the classified roads on a map by map basis and made corrections as thoroughly as possible by visually identifying errors or misclassified areas. This process involved comparing the classified roads with their counterparts from the original maps to ensure the road dataset aligned as closely as possible with the roads depicted in the maps. Although these manual corrections were simple manual edits to the shapefile and do not require specialized expertise, the process is highly time-consuming, offers limited possibilities for automation, and remains somewhat subjective depending on the individual carrying out the corrections. Considering these issues and the contextual inconsistencies in maps from different time periods and editions, there are likely some remaining topological inconsistencies that could lead to issues when using this dataset for routing or similar analyses.

Additional complexities emerged from other symbols on the maps. Other symbols crossing the roads often introduced small gaps in the road lines that the classifier did not recognize as roads. These gaps necessitated manual reworking and connection. Several inconsistent parts occasionally resulted in irregular and crooked road lines after converting the classification result to the road centre polyline. Other issues were induced by similar looking features on the maps, such as contour lines of similar line distances or other parallel line elements with a similar colour scheme, and led to false positive classifications of elements that were not actual roads. This may lead to an overestimation of available roads and needed to be corrected manually by identifying these predominantly unconnected line elements.

As it has been already stated in previous studies, achieving sufficient classification results relies on relatively large training datasets^34^. Selecting too few roads for training leads to poor classification outcomes. However, the time required for model calculation from the training data significantly increases with larger training datasets and depends on available computation power. In our case, the computational processes were carried out using a NVIDIA Quadro P4000 GPU and training times for each symbol ranged from several hours to two days, depending on the size of the training data. The classification times primarily depended on the number and image size of the maps being processed. In our case, each map required 5 to 10 minutes. This resulted in classification times of less than an hour for symbols present on only a few maps, but over a day for symbols appearing on more than several hundred maps. The time required for the conversion and merging of all classified roads was negligible. In total, the most significant factor influencing the overall effort and processing time is the number of distinct road symbols that need to be classified. The effort required is multiplied by the number of road symbols, as the steps 3 to 7 from Fig. 3 must be completed for each road symbol. The most time-consuming factor requiring human involvement was the creation of training data and post-processing. In particular the post processing took approximately 2 months in our case. The training, classification and conversion process was also computationally time-consuming, but required only minimal human interaction due to a high degree of automatization.

### Historical Road Development in Kenya and Temporal Data Inconsistencies

The development of roads in Kenya has been highly influenced by the country’s colonial past and individual political interests. The majority of Kenya’s mid 20th century road infrastructure consisted of predominantly unpaved roads, tracks, and trails of gravel and earth that serve as linkage of small rural villages and towns. Road development was highly biased towards linking urban centres and small towns during the colonial era to connect administrative headquarters and reduce travel time^42^. Thus, most attention was laid to developing the more densely populated areas of Kenya, mainly located in the southern part of the country. The first hard-surfaced roads were constructed in urban areas, beginning in Nairobi in the 1920s. Bitumen-surfaced roads were constructed in the other major towns during the 1930s^41^. In rural areas, roads remained little more than dirt tracks, maintained under forced labour, with local chiefs required to recruit local workers for the unpaid task. The work was not popular [^41^, pp. 30–32] and roads were widely viewed as symbols of colonial oppression.

The first great extensions of Kenya’s road system came in the 1940s and 1950s, partly stimulated by economic factors and partly by the demands of militarisation during the war and then the Mau Mau rebellion after 1952. Invariably conflict, and the need for security access, did more than anything else to promote improvements to the road network under colonialism [^41^, pp. 96–99]. In the 1950s, security problems allowed a number of military surveyors to be deployed on road works in Kenya, created greatly enhanced opportunities for aerial surveys, and brought funding for additional DOS staff to be assigned to the Survey of Kenya, all of which hugely assisted road construction as well as map making^51^. The extension of Kenya’s road network continued after independence in 1963. Roads became the ’gift of development’ for rural communities, improving communications and connecting them to markets. But this gift could be given, and also denied: from the 1970s, roads increasingly became entangled in the politics of preferment, as allocations for road construction and repair were determined by affiliation and association. Burgess *et al*.^50^ have shown that the roads became a major element in the ethnicization of Kenya’s politics over these years into the early 1990s. Regions with low levels of political leverage in government were unlikely to see road construction or repair. The classic example of underdevelopment in this regard is to be seen in Kenya’s north, where the Northern Frontier District (NFD) was placed under military administration from the early 1960s, and for the next 30 years saw only negligible infrastructural investment. The NFD was mainly considered as unprofitable due to the desert nature of the territory, the sparse population density, and people with nomadic habits that seemed impracticable for any form of native administration^58^. Thus, most attention was laid to developing the more densely populated areas of Kenya, mainly located in the southern part of the country. Those few roads that were developed in this period in the NFD were invariably for security access^59,60^.

The extracted historical road network of Kenya shown in Fig. 6 perfectly reflects these developments by showing a much denser road network in the southern parts of the country and an overall sparse road density in the northern areas. However, the fact that the road network has been extracted from maps spanning a time period of more than 30 years, leads to some limitations of the dataset in reflecting the state of Kenya’s roads at a certain time period. Each road only represents a snapshot in time corresponding to the year of the map available for that location and as the temporal distance from this point increases, so does the uncertainty regarding the state or existence of the road. The level of uncertainty regarding the existence of roads varies depending on the temporal perspective of the road dataset. This uncertainty increases at both ends of the time period covered by the study, particularly as the classified map dates further from the study’s time frame. It is assumed that all roads classified likely existed in the 1980s. However, for older maps, there is growing uncertainty that additional roads existed in the 1980s but are missing from the map, potentially underestimating the road network for that period. Conversely, for newer maps, uncertainty increases about whether a road already existed in the 1950s, possibly leading to an overestimation of the road network for earlier periods. While these uncertainties can be estimated for maps with known creation years, they cannot be accurately determined for maps with unknown dates.

Additionally, the historical road development of the country at that period shows that the degree of uncertainty in road presence or absence in the extracted dataset varies significantly across regions. In northern Kenya, road infrastructure experienced only minimal development during the 1950s–1980s leading to a high likelihood that the overall road network remained relatively unchanged throughout the period covered by the presented dataset. Conversely, several regions in southern Kenya underwent substantial road development during this era, leading to a higher degree of uncertainty about road presence and state in those areas. However, the absence of suitable reference datasets prevents a more detailed assessment of these potential temporal inconsistencies in the extracted historical road network of Kenya.

## Usage Notes

In this study, we have extracted historical road information from more than 500 digitized topographic maps of 1:50,000 and 1:100,000 scale. By extracting the historical road network from these maps, a detailed road network covering the time period between the 1950s and 1980s could be created that was previously unavailable for Kenya. Making this historical information available provides opportunities for long-term research about the evolution of infrastructure and its effects on Kenya’s landscape and land use changes over time. The extracted historical road network dataset will support future research within the CRC/TRR228, but also other studies related to socio-economic and ecological topics in Kenya. The successful application of deep learning methods allowed the transformation of geographic information from analogue maps into digitally usable formats and making them valuable for future research. This approach shows an applicable method to map historical road networks on a national-level scale. Moreover, these feature extraction techniques are likely transferable to digitize and extract other geographical information printed on maps, such as railways, land cover and vegetation features, or human settlements, provided there is sufficient coverage with suitable topographic maps of an appropriate scan quality.

## Data Availability

The study has been conducted using ESRI’s ArcGIS Pro Software tools for deep learning model training, classification, and vector data conversion in two Python scripts that are accessible in the data repository of the CRC/TRR228 at: 10.5880/TRR228DB.32^54^. Please note: A commercial ArcGIS Pro license is required to run the scripts.
